# Teaching the Fundamentals of Biological Data Integration Using Classroom Games

**DOI:** 10.1371/journal.pcbi.1002789

**Published:** 2012-12-27

**Authors:** Maria Victoria Schneider, Rafael C. Jimenez

**Affiliations:** 1 Outreach and Training Team, European Molecular Biology Laboratory Outstation, European Bioinformatics Institute, Wellcome Trust Genome Campus, Hinxton, United Kingdom; 2 Proteomics Services, European Molecular Biology Laboratory Outstation, European Bioinformatics Institute, Wellcome Trust Genome Campus, Hinxton, United Kingdom; Whitehead Institute, United States of America

## Abstract

This article aims to introduce the nature of data integration to life scientists. Generally, the subject of data integration is not discussed outside the field of computational science and is not covered in any detail, or even neglected, when teaching/training trainees. End users (hereby defined as wet-lab trainees, clinicians, lab researchers) will mostly interact with bioinformatics resources and tools through web interfaces that mask the user from the data integration processes. However, the lack of formal training or acquaintance with even simple database concepts and terminology often results in a real obstacle to the full comprehension of the resources and tools the end users wish to access. Understanding how data integration works is fundamental to empowering trainees to see the limitations as well as the possibilities when exploring, retrieving, and analysing biological data from databases. Here we introduce a game-based learning activity for training/teaching the topic of data integration that trainers/educators can adopt and adapt for their classroom. In particular we provide an example using DAS (Distributed Annotation Systems) as a method for data integration.

## Why and How Data Integration Is Useful

Data integration gives scientists a view of the bigger picture surrounding their experiments and allows them to make better and faster decisions about their research. Examples of data integration enabling biological discovery include expression data combined with pathway information to analyse changes in metabolic and signalling processes in cancer diseases [1], identification of functional elements and regulatory circuits in *Drosophila* [2], understanding protein disorder through comparative genomics and genetic interactions [3], as well as the efforts in integrating data from a variety of levels (and types) across pathways [4]. Having a better understanding of data integration and its application to bioinformatics can help trainees, bioinformaticians, and software engineers to make the most of their data. Good integration facilitates data sharing between labs, resulting in decreased costs of unnecessary duplication of experiments. Integration of multiple data sources helps increase confidence in results if consensus is shown by different experiments.

In biological research, data integration can mean reading papers so you can write a report, exploring database websites so you can learn about a topic, or downloading some data from different databases so you can analyse them. Such tasks have become, given the advent of high throughput technologies, increasingly necessary and more challenging. Ultimately, when researchers produce data, they face the need to be able to download some data from different databases and combine them with their own data. Good examples are the efforts in systems biology where data that originate from different experimental technologies/methodologies are integrated to provide a larger and more complete picture of a system [5]. Existing biological databases are distributed across the Internet. Biological data are often complex and heterogeneous (different formats and data types), and it is challenging to retrieve them, map objects, and make them accessible in an integrated and flexible manner [6]. It is obvious that anyone dealing with such vast amounts of data needs to rely on data matching and integration tools, minimizing manual inspection and quality control. This involves using computers to automatically pull in data from different locations, process them, and create a resource derived from the data. Data integration, however, is far from trivial, especially within molecular biology where there are vast and growing numbers of databases [7], each with a specific query interface to search and retrieve the data. Numerous and diverse query interfaces combined with a lack of a central registry describing databases hinders the ability of scientists to find the data relevant to their research. Different databases provide results in different formats using different data structures and vocabularies, acting as obstacles to data integration. As a result, diverse data integration strategies have been adopted in bioinformatics to combine information residing in different sources. There are several articles describing data integration and the methodologies that they present [8]–[13].

## Current Approaches to Data Integration

**Figure 1:**
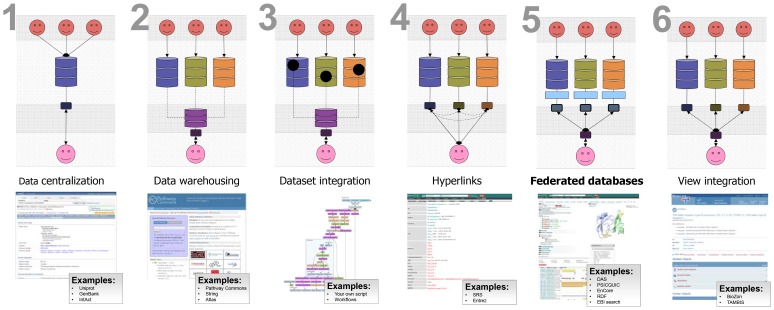
Shows popular data integration approaches, with examples, for resources that have implemented these approaches.

Data integration sounds like a simple idea, however it is not an easy topic to introduce. It is a generic topic that is subject to many interpretations. Generally, data integration can be defined as the process of combining data residing in diverse sources to provide users with a comprehensive view of such data. There is no universal approach to data integration, and many techniques are still evolving (as shown in Figure 1).

Though many efforts on data integration have been made, there are still major problems to solve. Data integration was recognized by Karp [14] as adding value to biological discovery, and although less than a decade ago there was some scepticism about the feasibility of the process [15], [16], several efforts in computational biology have been successful (Table 1).

**Table 1:** Some of the major successful and recognized examples of data integration.

System and Key Reference	Description	Data Types
Biomart [27]	Federated database system that provides unified access to disparate distributed data sources	Genome, gene annotation, protein sequence, protein structure, pathways, gene expression, protein identifications
DAS [18]	Client-server system in which a single client integrates information from multiple servers.	Genome, gene annotation, protein sequence, protein structure, molecular interactions, gene expression, protein identifications
SRS [34]	SRS is a data integration, analysis, and display tool for bioinformatics, genomic, and related data	Genome, gene annotation, protein sequence, protein structure, molecular interactions, pathways, gene expression
Ondex [35]	The Ondex data integration platform enables data from diverse biological data sets to be linked, integrated, and visualised through graph analysis techniques.	Genome, gene annotation, protein sequence, protein structure, pathways, gene expression
Bio2RDF [29]	Integrates diverse biological information and enables powerful queries across multiple data types using semantic data integration techniques	Genome, gene annotation, protein sequence, protein structure, pathways, chemical compounds
InterMine (http://intermine.org)	InterMine is used to create databases from a single data set or integrating multiple sources of data; it is used specially for model organism database	Genome, gene annotation, protein sequence, protein structure, molecular interactions, pathways, gene expression, protein expression, metabolites

From our experience, however, when training life scientists with no computational background, it often transpires that there is a lack of real understanding at various levels (e.g., what integrating data from different resources means, which assumptions are made, where the data originate or reside). This article does not aim to make the reader an expert on data integration. It aims to introduce in an accessible and problem-solving manner the concepts underlying data integration, the terminology used, and the challenges involved. Specifically, it targets trainers and educators by providing ways (in the form of a game-based learning activity) in which the concepts of data integration can be introduce also to a non-computational audience.

## Guide for Teaching about Data Integration

The following section is a guide on how to teach data integration in face-to-face events. We have tried this in audiences ranging from 6 to 40 attendees.

1. The first step is to ensure trainees understand the Learning Outcomes and that the expectations of what this session is about are stated from the start. Often, this session demystifies the misconceptions of a “magical” solution where bench trainees could simply press one button on a web page and get a digested set of integrated data. Understanding the challenges of data integration is crucial and can help ensure that life scientists (a) appreciate the importance of submitting their data to repositories and (b) do so in accordance with the community standards. The latter, however, represents a challenge in its own right [17]. Therefore, we concentrate on four main Learning Outcomes, which should be made explicit to the audience, in which we say, “by the end of this session you would be able to”:
  (i) Describe the major challenges in data integration;
  (ii) Understand the source of the data as well as the algorithm used for combining data;
  (iii) List the components you need to ensure data integration;
  (iv) Describe the major components used to integrate genome annotation data and how a typical query actually works.
2. Consequently, the actual terminology and definitions related to data integration would be introduced and clarified.
3. Once this is done, trainees will be explained the fact that they will use the “new” terminology to play a game that will allow them to understand how the different parts work and ultimately how data integration takes place.
4. Once the game is played, there will be an opportunity to revisit the concepts. This can be done as an open discussion, or separating the trainees into groups and asking them to summarise (i) one thing they felt they learned, (ii one thing they feel puzzled by, and (iii) how data integration and the concepts covered in this session relate to the queries and data analysis they are trying to do. This can be done by asking each group to summarise these on flipchart paper and to share this with the rest of the class. Ultimately, this part will often depend on how much time is available. We recommend at least 30 min for this session, although the session would benefit from extra time for reflection and discussion, if practicable.

The rest of the article will cover in detail points 2 and 3. The final part of the article describes how the materials developed for the specific method called “Distributed Annotation Systems” (referred throughout the article by its abbreviation DAS) have been successfully used in training a variety of trainees with different backgrounds.

### How Automatic Data Integration Works

In order to fully understand data integration and bridge the technical aspects for a biological audience, it is important to clarify some basic concepts as well as to become acquainted with the terminology. This section will introduce concepts such as distributed data, service-oriented architecture, web services, data standards, and the client-server model, illustrating an example for the federated model. We will use genome annotation as an example as we introduce the various concepts and will use a federated model (DAS) to describe a genome-level example. The example will be contextualised in order to make explicit the relevance of such concepts for resolving a biological problem/question. A summary list with key terminologies and definitions can be found in Box 1.

**Box 1. List of Useful Key Terminology and their Definitions**

**Data integration:** the process of combining data that reside in different sources, to provide users with a unified view of such data.

**Distributed data:** literally intended as the data not being in a single centralised repository but distributed. This also applies to different types of data or levels (e.g., raw sequences in one place, annotation of these sequences stored in another place).

**Client service model:** when the tasks and workload are distributed between the providers of a resource or service (called servers) and service requesters (called clients).

**Federated model:** when the constituent databases are interconnected via a computer.

**Client:** the service requesters (e.g., Ensembl is the client, the request being: annotation from).

**Server:** the providers of resources (e.g., raw sequence data) or service (e.g., a specific annotation).

**Source:** databases that have adopted the DAS rules. It is important to distinguish that “Source” is presented here as the database or informatics resource from which the data are obtained. However, databases are the repositories of experimental or inferential data obtained as part of the work of scientists. Most databases explicitly state where the data originate from and how these data are generated (e.g., manual/automatically inferred).

**Registry:** the list of sources.

**Annotation:** enrichment with information of raw biological sequence.

**XML:** stands for eXtensible Markup Language and is a set of rules for encoding documents in machine-readable form.

**Webservice:** software that runs remotely, which is accessible over a network (e.g., the Internet) and is meant for machine-to-machine communication.

**DAS:** the Distributed Annotation System (DAS) defines a communication protocol used to exchange annotations on genomic or protein sequences.

**Service Oriented Architecture (SOA):** flexible set of design principles used during software development that defines how a loosely integrated suite of services can interface among themselves and be used within multiple business domains.

#### Distributed data

Distributed data simply means that data are not kept in one place but are shared [18]. In 2001 Dowell et al. [19] described how genome annotation, curated by centralized groups with limited resources and efforts to share annotations transparently among multiple groups, did not work. As a solution to this problem, Dowell et al. presented the DAS. DAS allows sequence annotations to be decentralized among multiple third-party annotators (e.g., Ensembl, UniProt, InterPro, UCSC, CBS) and then be integrated on an as-needed basis by the client-side software (some examples of genome clients are Ensembl, Gbrowse, Dalliance, IGB). The client and server “communicate” by a protocol following the same XML specification (XML stands for eXtensible Markup Language and is a set of rules for encoding documents in machine-readable form). Annotations are displayed in layers (e.g., Transcript from Cosmic, genetic phenotypes from OMIM, Genome reference information from Ensembl, gene annotation from UCSC, Copy-number variations from University of Toronto, etc.), one per server. Any client or server adhering to the DAS XML specification can participate in the system. At the time of writing this article, the DAS registry [20] provided 1,848 DAS sources, of which 1,083 were genome annotation sources.

#### Service oriented architecture

Service-oriented architecture (SOA) is an approach for organizing software in the form of independent, interoperable services that can be composed and recomposed to meet multiple requirements [21]. In the case of genome annotation, this translates into Genome clients to visualize genome data, a collection of sources providing genome annotations (SNPs, CNV, phenotypes, etc.) and a registry to list sources.

#### Web service

A standard technology that allows interoperability among computers to exchange data [22]. In simple terms, a web service is software that runs remotely, which is accessible over a network (e.g., the Internet) and is meant for machine-to-machine communication. It is independent from programming languages and can be operated following specific rules. The technology is built mainly on two open standard protocols: REST (Representational State Transfer) and SOAP (Simple Object Access Protocol).

#### Data standards

The standardization of how to report data is a priority [23]. Enabling the integration of data generated in laboratories across the globe is recognized as a promising way to revolutionize biology [24]. The need to develop standards has already influenced genomics and transcriptomics. Projects that have previously been viewed as being too big to implement can now be distributed across multiple sites [25]. Public databases for gene sequences, transcriptomics, and proteomic experiments are now available, and similar efforts for many other fields are currently ongoing. For our case study on genome annotations, a clear and successful example is represented by the Gene Ontology [26]. Ontologies in the bioinformatics community seek agreement to define biological concepts and its relationships. The adoption of a common terminology facilitates the effective combination of data from multiple heterogeneous sources.

#### Client-server model

This model is a distributed application that partitions tasks or workloads between the providers of a resource or service [27], called servers (Ensembl, UniProt, UCSC, InterPro, CBS), and service requesters, called clients (Ensembl, Gbrowse, Dalliance, IGB). For our example on genome annotation, a typical client-server model would be DAS. Data distribution, performed by servers, is separated from visualization, which is done by clients.

#### Federated model

**Figure 2:**
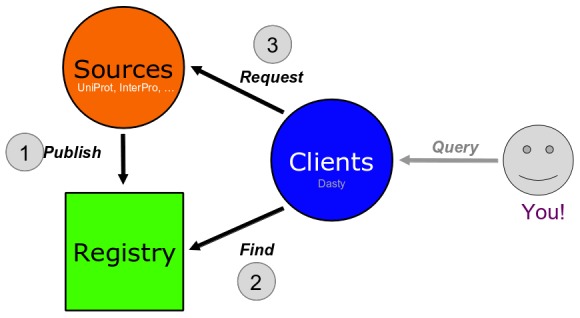
Schema of the four major roles in data integration in a federated system.

Our definition of a federated model is that the constituent databases are interconnected via a computer network. Data from multiple autonomous databases (e.g., CNV data from the University of Toronto and phenotype information from OMIM) are transparently integrated into a single federated system providing a single entry point to query and retrieve genomic data (Figure 2). Other examples of federated models besides DAS are Biomart [27] and PSICQUIC [28].

Though we provide a definition for the specific terminology, it is important to appreciate that the trainees do not need to know in advance this terminology. By playing the game, they will get familiar with these concepts and associated terminology at the end of the game. Using DAS as a model for teaching/training data integration to a non-expert audience, we present an example for the case of genome annotation in the following section.

### Learning by Playing a Game: DAS as a Case Study

The use of educational games within learning environments raises motivation, increases interest in the subject matter, intensifies information retention, encourages collaboration, and improves problem-solving skills [29]. Educational games can be used as a tool at different stages within the learning process: at the beginning of the lesson as a motivation element, during the main lesson to complement other activities, or at the end as revision or remedial teaching. Games in bioinformatics education can help trainees to understand technical computational concepts more intuitively and bridge the cultural gap between computational and experimental scientists. Games in data integration aid trainees in their understanding of technical computational concepts, biological concepts, as well as how to use bioinformatics to solve a problem. Several studies have shown the effectiveness of games for educational purposes [30]–[32], though to our knowledge this is the first application for introducing trainees to computational data integration.

The DAS game is an example of how to introduce trainees (from a variety of backgrounds: life scientists to bioinformaticians to high school teachers) to data integration of genome and protein sequence annotation. The DAS defines a communication protocol motivated by the idea that such annotations should be provided by the source resource and not from a one-off centralized resource. The DAS game is easily adoptable in the classroom where the trainees become actual parts of the integration system (e.g., user, client, registry, reference source, and annotation sources). The query is the main biological question/problem the trainees are requested to solve by applying what they learn about integration and DAS.

By applying the rationale described in the sections above, the trainees will be prompted to devise workflows in order to achieve sensible and meaningful data integration that would provide information to solve specific biological problems and tasks. The ultimate aim of this section is to recapitulate the concepts and skills explained throughout the article so that the trainee learns in an informative way not only how to “plug in” the latest data (including users' own data) but also what this means and when it actually makes sense to do this.

The game is intended for a wide audience, including life scientists, bioinformaticians, and software engineers. For life scientists, the aim is to give them a better understanding of data integration, and an introduction to the field of bioinformatics. For bioinformaticians, the aim is to make them aware of the different strategies and ideas about data integration as well as to provide an opportunity to improve their data integration skills. For software engineers working with scientific data, the aim is to get them familiar with tools used in bioinformatics and give an overview of status and further directions in data integration in bioinformatics. Providing some tuning for the level of background information and jargon (that can be done by the trainer in the introduction to the game), we have also implemented the game for high school teachers and undergraduate-level courses.

### How to Play the Game

What trainees need to know before getting started:

1. They do NOT need any prior knowledge of bioinformatics.
2. They need to know what nucleotide and gene sequences are, and what annotation is.

The game needs at least six players, and we recommend no more than 40. Each person will get a card representing one role (see figures following the activity).

What to do:

1. Distribute cards defined as one of the following categories: User, Client, Sources, Registry. You can find the latest version of the cards for this game in the Bioinformatics Training Network: http://www.biotnet.org/training-materials/das-game.
2. Explain roles.
3. Describe each type of cards.
4. Play the game until everyone gets it.

A step-by-step guide for the game can be found in Box 2.

**Box 2. The DAS Game: Step-by-Step Guide**

1. Get your sources registered
  - Ask all the Sources to register in the Registry.
  - Sources should provide the following information:
    - Name
    - Type
    - Function (e.g., sequence only? features only? sequence and features?)
    - Location.
  - Ask the Registry to write down this information.
2. Identify the main players (play through from this point once for genomes, then once for proteins)
3.
  - Ask the genome Client to come forward
  - Ask the genome User to show his or her card
4. The User queries the Client
  - Ask the User to choose a query from the list.
  - The User should send the request to the Client.
  - Ask the Client to write down the query in the following format:
  -
    1. ID:start,stop (where “ID” is the identification number, “start” is the first entry on the list, and “stop” is the last)
5. The client retrieves sequence information
  - Find a genome reference Source.
  - The Client should ask the Registry if it knows of any Source providing nucleotide **sequences**.
  - The Client should obtain the name and location of the genome reference Source.
6. Request sequence
  - The Client should ask the genome reference Source if there is any sequence for a given ID, and if so, the Client should request the specific range they are looking for.
  1. The genome reference server dictates the sequence.
  2. The Client writes the sequence on the board.
7. The client retrieves features (annotations)
  - Find genome annotation Sources.
  - The Client should ask the Registry if it knows of any Source providing genome **features**.
  - The Client should obtain a list of names and locations of genome annotation Sources.
  1. Ask the client to list these sources.
8. Request annotations
  - The Client should ask each listed genome Source if it has any features for the requested ID. If so, the Client should request features for the specific range they're looking for.
  - Each annotation server dictates the name of the feature and its range.
  - The Client draws the dictated features on the board, below the sequence.

#### The roles

Sources are databases that have adopted the DAS rules. To let everybody know they exist, sources make sure they appear in a registry. Sources tell the registry (1) where they are located and (2) what type of information they provide. A client is software that has been developed specifically to combine information from different sources. At a user's request, a client contacts the registry to find out what sources are available and where they are located. Then, the client queries all the sources, retrieves data, and presents them in a unified way (Figure 2).

#### Source

A source is an individual database (e.g., UniProt, Ensembl). A source is defined by the type of information it provides (e.g., proteins or genomes). It can also be defined by what it can do: it might provide annotation (additional information about a sequence such as associated coding regions, structural RNA, variation information, exon, introns, etc.), or it might provide only sequences.

In this exercise, we refer to “annotation” sources and “reference” sources. A DAS is made up of one or more annotation sources, each of which refers to a reference source.

Annotations are usually “positional”: they refer to a specific location within a sequence. A protein domain within a protein sequence is an example of such an annotation.

Annotations can also be “non-positional”: for example, a description of a protein is an annotation that is attributed to a whole sequence.

**Figure 3:**
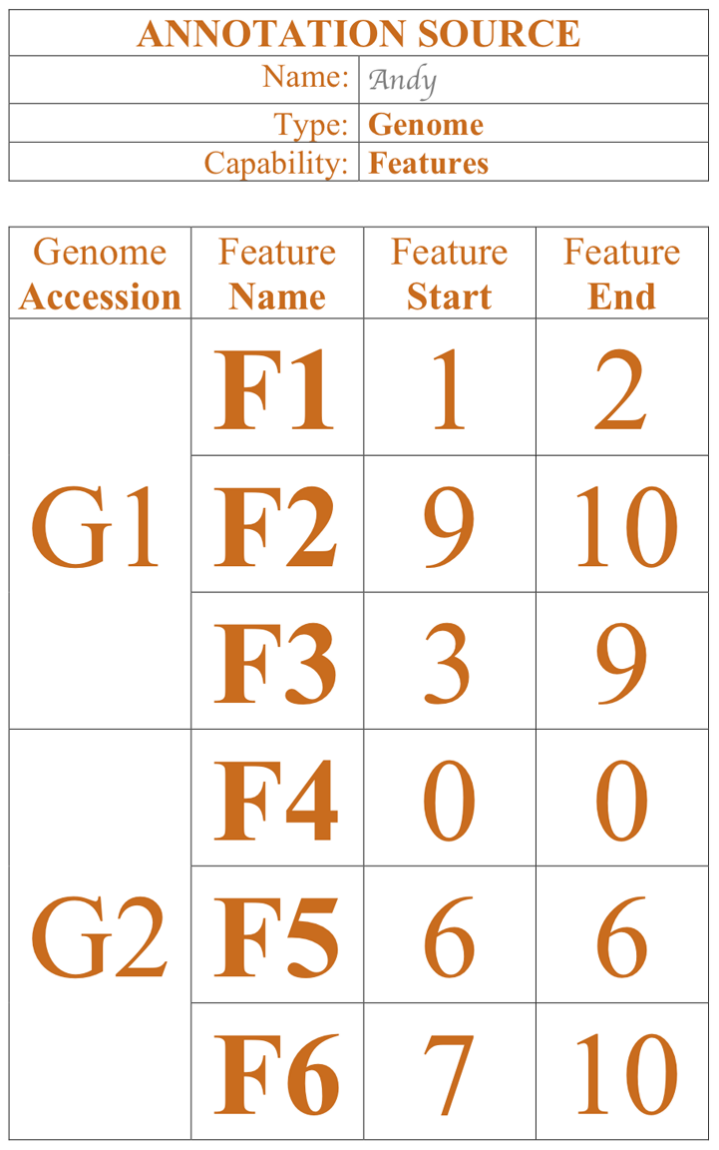
Example of a genome reference source representing a database including a list of genome features.

**Figure 4:**
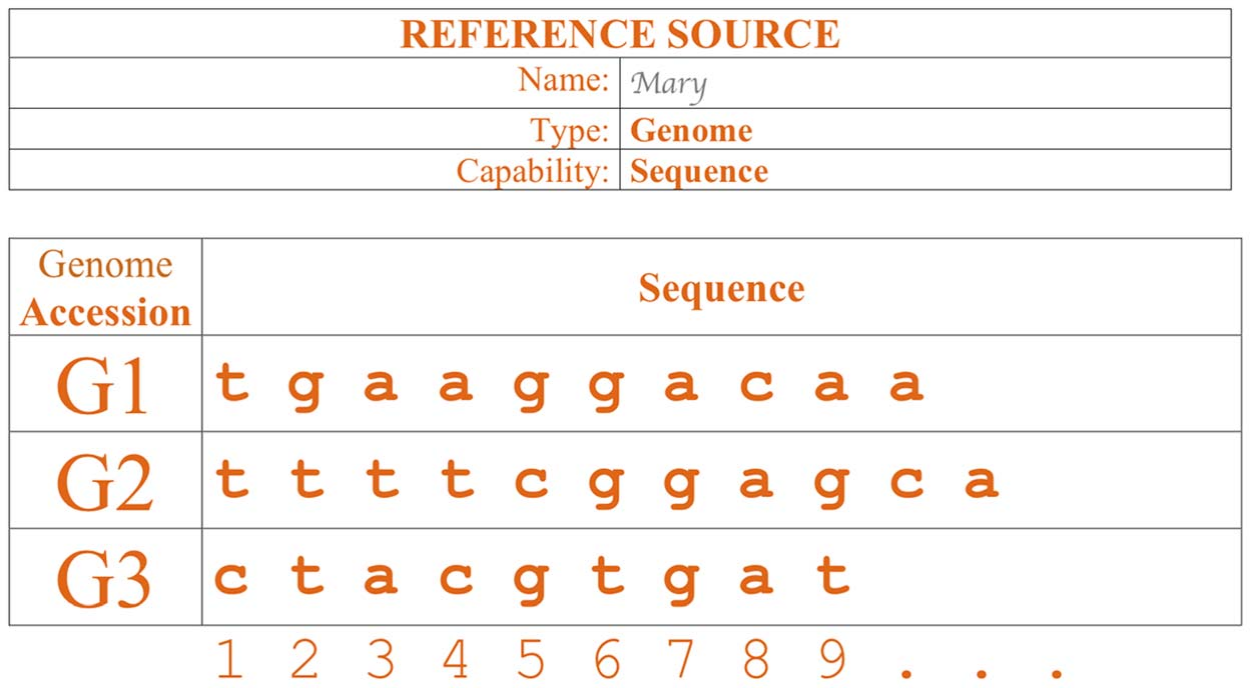
Example of a genome reference source representing a database including a list of nucleotide sequences.

Example of a genome annotation source (Figure 3):

- A database with genome information containing three annotations (“features”) for a nucleotide sequence named “G1” and three annotations for a different nucleotide sequence named “G2.”
- A database with genome information containing annotation (“features”) for two nucleotide sequences named “G1” and “G2.”
- The annotations named for G1 are “F1,” “F2,” “F3,” and the annotation for G2 are “F4,” “F5,” and “F6.”
- Annotation “F1,” “F2,” “F3,” “F5,” and “F6” are positional. “F6” is located between G1's nucleotide 7 and nucleotide 10.
- “F4” is non-positional; to indicate this aspect, its feature range is marked as 0 to 0.
- A database with three nucleotide sequences, named “G1,” “G2,” and “G3.” “G1” is a sequence of 10 nucleotides: “t g a a g g a c a a” (Figure 4).

#### Registry

**Figure 5:**
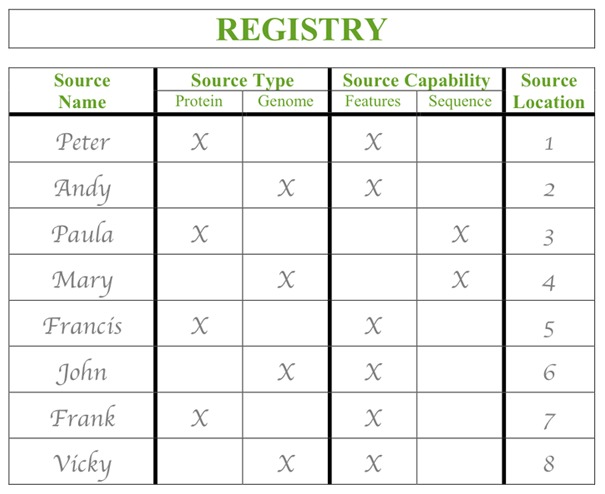
Example of the card for the “Registry” after genome sources have been registered.

Like a telephone book, the registry is a list of sources (see an illustration of a registry in Figure 5). The registry collects important information about sources like the “name,” “type,” “capability,” and “location.” This will allow clients and users to easily find specific sources. The source has the responsibility to ensure that it is listed in the registry by registering with it.

#### Client

**Figure 6:**
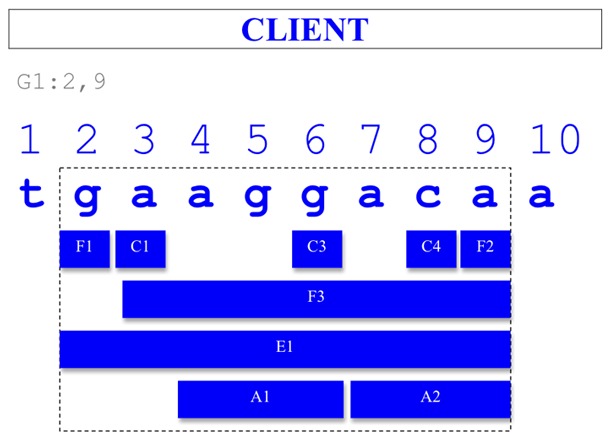
Example of the graphical representation someone playing the “Client” role could come up with.

A client is a tool (normally a graphical user interface, or GUI) that integrates data from different sources. The client responds to a user query to provide a unified view of information. An example is illustrated in Figure 6.

#### User

**Figure 7:**
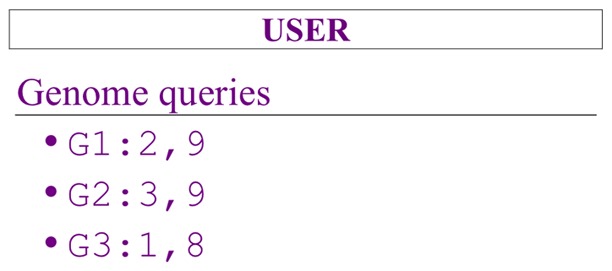
Game card used to play the role of the “User” including a selection of genome queries.

As the name suggests, this is the person using a Client to get sequence information. For instance, the user could request genome information within a range of nucleotides. For instance, “G1:2,9” is a request for any information (sequences and features) for “G1” between the nucleotide “2” and “9.” Figure 7 illustrates an example of a User.

**Figure 8:**
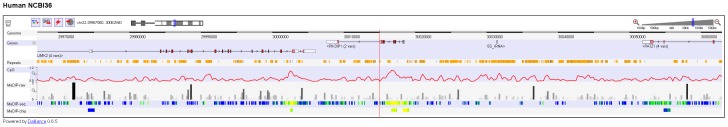
Example of a DAS genome client (Dalliance) displaying positional annotations from several sources (e.g., Ensembl, HGNC, Agilent probesets, and Toronto CNVs) [**33**].

Using a DAS client (Dalliance [33], for example), the User queries for genome information, which prompts the Client to use the Registry to identify and query the relevant Sources. In Dalliance each track represents data from a DAS source. Figure 8 illustrates Dalliance as the DAS genome client (Dalliance) displaying positional annotations from several sources.

Box 2 provides a quick guide to the DAS game step-by-step.

## Results from Prior Teaching of This Material

The main outcome from this has been that trainees learned key concepts underlying one of the common strategies used in data integration—in an active and engaged manner and all without a computer. Trainees also learn the basic terminology and experience some of the challenges in the field of data integration. The activity ultimately illustrates to trainees how they can use bioinformatics to solve a problem. This also helps the non-computational audiences to contextualize the issues they had in understanding limitations of data integration and encourages better communication and cross-talk between computational and non-computational scientists. The Game has been a success in a variety of life science courses targeting different audiences: researchers, bioinformaticians, developers, and teachers. It has been played as part of introductory courses on proteomics and bioinformatics (e.g., WT-EBI Proteomics Bioinformatics Course 2011) and in training courses to teach bioinformatics to science teachers as part of the ELLS programme (http://www.embl.de/training/scienceforschools/teacher_training/learninglabs/index.html). We also had positive feedback when the game was used in more technical events like the DAS workshop (http://www.biodas.org/wiki/DASWorkshop2011) or the EBI databases programmatic access course.

The reaction of the trainee is always very positive; they enjoy team work and they like to be actively engaged. They have fun and naturally learn by playing. The success of the game depends on the participation of all the trainees who play different roles. This encourages every trainee to pay attention and intuitively understand the concepts while playing. At the end of the game, trainees get a good understanding of how data federation works and how to apply data integration concepts to manage molecular biological data. The trainee will gain different skills depending on the target audience: bench biologists learn how the technology works and how to use it to access the data, developers gain exposure to biological concepts and learn how to use bioinformatics to manage biological data, bioinformaticians benefit from all of these things, and teachers in the life sciences (of undergraduate level and below) gain a good introduction to how useful (and essential) bioinformatics is in life science research and in future progress.

The game is not just useful for trainees but also for the trainers and educators, who find in the game a useful tool to reinforce concepts and break the ice. The DAS game was originally designed as response to the feedback obtained in the DAS workshops. In the past, trainees attending the DAS workshops showed difficulty with assimilating all the concepts related to data integration due to the amount of technicalities described in a short period of time. The game helped us to clarify these concepts from the very beginning and made the subsequent workshops that included the game more productive.

The game including cards and instructions is available on the BTN website (a community-based project providing a centralised facility to share training materials) with other training material about DAS: http://www.biotnet.org/search/node/das. Text S1 provides a syllabus for the DAS game.

## Supporting Information

Text S1 Syllabus for the DAS game presenting the learning aim and objectives, references for background information, instructions and rules of the game, assessment criteria, and attendance requirements.
(DOC)
