# Supplementary material for: Teaching the Fundamentals of Biological Data Integration Using Classroom Games
Source: PLoS Comput Biol. 2012 Dec 27;8(12):e1002789. doi: 10.1371/journal.pcbi.1002789 (PMC3531283; doi:10.1371/journal.pcbi.1002789)
Supplement: Text S1 — Syllabus for the DAS game presenting the learning aim and objectives, references for background information, instructions and rules of the game, assessment criteria, and attendance requirements. (DOC) [file pcbi.1002789.s001.doc]

**Supporting Materials accompanying the paper entitle “Teaching the fundamentals of biological data integration using classroom games”**

**COURSE NAME**: Introduction to Biological Data Integrations: what really happens behind the screen...

**DURATION:** minimum 30min, depending on the audience this can be longer (e.g. in case more background information is necessary).

**PREREQUISITES:** What trainees need to know before getting started:

(1) They do NOT need any prior knowledge of bioinformatics

(2) They need to know what nucleotide and gene sequences are, and what annotation is.

**EQUIPMENT:** Each group of trainees will need a selection of cards, one for each person (see materials section below).

**LEARNING AIMS:**
This course is designed to give trainees an immersive experience of how biological data can be integrated.  By asking trainees to interact through a game activity representing the various parts of the computational data integration progress the course also seeks to provide trainees with an easy way into the specific terminology and the concepts involved. Trainees work on a variety of levels: speaking, listening comprehension, reading and interactively playing the game.

**LEARNING OBJECTIVES**

Upon completing this course, trainees will possess the ability to:

- Describe at least three main challenges in biological data integration.
- Explain what is the source of the data, the difference between where the data originates or resides.
- List the components you need to ensure data integration in a federated system (registry, client, user...).
- Describe the major components used to integrate genome annotation data and how a typical query actually works.
- Describe the flow of communication among the different components.
- Explain why data standards are important in data integration.
- Explain the separation between client and server.
- Describe how to query, retrieve and integrate information in a federated system.
- Describe how the DAS system works.

**BACKGROUND INFORMATION ON DATA INTEGRATION**

There are several seminal papers and reviews on biological data integration. Below some suggestions to further obtained more background information on the topic of data integration:

- Amit Sheth, James Larson. 1990 "Federated database systems for managing distributed, heterogeneous, and autonomous databases". ACM Computing Surveys, Vol. 22, No. 3, September 1990.
- Olga Brazhnik and John F. Jones. 2007. Anatomy of Data Integration. J Biomed Inform. 2007 June; 40(3): 252–269.
- Wong L. 2002 Technologies for integrating biological data. Brief Bioinform. 3(4):389-404.
- Achard F, Vaysseix G, Barillot E. 2001 XML, bioinformatics and data integration, Bioinformatics. 17(2):115-25.
- Joyce AR, Palsson BØ. 2006 The model organism as a system: integrating 'omics' data sets. Nat Rev Mol Cell Biol. 7(3):198-210.
- Hwang D, Rust AG, Ramsey S, Smith JJ, Leslie DM, Weston AD, de Atauri P, Aitchison JD, Hood L, Siegel AF, Bolouri H. 2005. A data integration methodology for systems biology. Proc Natl Acad Sci U S A. 102(48):17296-301.
- Letunic I, Copley RR, Schmidt S, Ciccarelli FD, Doerks T, Schultz J, Ponting CP, Bork P. 2004. SMART 4.0: towards genomic data integration. Nucleic Acids Res. 1;32(Database issue):D142-4.
- Karp P.D. 1996 Database links are a foundation for interoperability, Trends in Biotechnology, Volume 14, Issue 8, 273-279.

Importantly, there is no need to read any of the above papers in order to follow and comprehend the concepts and terminology covered by this course.

**MATERIALS**

As a trainer you can decide to explain the challenges in data integration, basic concepts and components in your preferred method and tuned to the audience you have to train.

You can find the ppt we used to introduce the actual game and all the various hands outs here: <http://www.biotnet.org/training-materials/das-game>.

**GAME SET UP INSTRUCTIONS**

Start by:

1. Distribute cards defined as one of the following categories: User, Client, Sources, Registry.

2. Explain roles.

3. Describe each type of cards.

4. Play the game until everyone gets it.

**The roles**

Sources are databases that have adopted the DAS rules. To let everybody know they exist, sources make sure they appear in a registry. Sources tell the registry (1) where they are located and (2) what type of information they provide. A client is software that has been developed specifically to combine information from different sources. At a user’s request, a client contacts the registry to find out what sources are available, and where they are located. Then, the client queries all the sources, retrieves data and presents it in a unified way.

**Source**

A source is an individual database (e.g. UniProt, Ensembl). A source is defined by the type of information it provides (e.g. proteins or genomes). It can also be defined by what it can do: it might provide annotation (additional information about a sequence), or it might provide only sequences. In this exercise, we refer to ‘annotation’ sources and ‘reference’ sources. A Distributed Annotation System (DAS) is made up of one or more annotation sources, each of which refers to a reference source. Annotations are usually “positional”: they refer to a specific location within a sequence. A protein domain within a protein sequence is an example of such an annotation. Annotations can also be “non-positional”: for example, a description of a protein is an annotation that is attributed to a whole sequence.

Example of a protein annotation source:

● A database with protein information containing four annotations (“features") for a protein named “G1”.

● The annotations are named “F1”, “F2”, “F3” , “F4” , “F5” and “F6”.

● Annotation “F1”, “F2” , “F3” , “F5” and “F6” are positional. “F6” is located between G1’s nucleotide 7 and nucleotide 10.

● “F4” is non-positional; to indicate this aspect, its feature range is marked as 0 to 0.

● A database with three protein sequences, named “G1”, “G2” and “G3”.

“G1” is a sequence of 10 nucleotides: “t g a a g g a c a a”

**Registry**

Like a telephone book, the registry is a list of sources (see an illustration of a registry in Figure 5). The registry collects important information about sources like the "name", "type", "capability" and "location". This will allow clients and users to easily find specific sources. The source has the responsibility to ensure that it is listed in the registry by registering with it.

**Client**

A client is a tool (normally a graphical user interface, or GUI) that integrates data from different sources. The client responds to a user query to provide a unified view of information.

**User**

As the name suggests, this is the person using a Client to get sequence informationFor instance the user could request protein information within a rage of amino-acids. For instance “G1:2,9” is a request for any information (sequences and features) for “G1” between the nucleotide “2” and “9”.

Using a DAS client (Dalliance for example), the User queries for genome information, which prompts the Client to use the Registry to identify and query the relevant Sources. In Dalliance each track represents data from a DAS source.

**GAME RULES**

**1. Get your sources registered**

1. Ask all the Sources to register in the Registry.
2. Sources should provide the following information:
   1. Name
   2. Type
   3. Function (e.g., sequence only? features only? sequence and features?)
   4. Location.
3. Ask the Registry to write down this information.

**2. Identify the main players (play through from this point once for genomes, then once for proteins)**

- Ask the genome Client to come forward
- Ask the genome User to show his or her card

**3. The User queries the Client**

- Ask the User to choose a query from the list.
- The User should send the request to the Client.
- Ask the Client to write down the query in the following format:
  - ID:start,stop (where ‘ID’ is the identification number, ‘start’ is the first entry on the list and ‘stop’ is the last)

**4. The client retrieves sequence information**

- Find a genome reference Source
- The Client should ask the Registry if it knows of any Source providing protein **sequences**.
- The Client should obtain the name and location of the protein reference Source.

**5. Request sequence**

- The Client should ask the genome reference Source if there is any sequence for a given ID and, if so, the Client should request the specific range they are looking for.
  - The genome reference server dictates the sequence
  - The Client writes the sequence on the board.

**6. The client retrieves features (annotations)**

- Find genome annotation Sources
- The Client should ask the Registry if it knows of any Source providing protein **features**.
- The Client should obtain a list of names and locations of genome annotation Sources.
  - Ask the client to list these sources.

**7. Request annotations**

- The Client should ask each listed genome Source if it has any features for the requested ID. If so, the Client should request features for the specific range they’re looking for.
- Each annotation server dictates the name of the feature and its range.
- The Client draws the dictated features on the board, below the sequence.

**ASSESSMENT CRITERIA:**
We do not define any assessment criteria; however those adopting this syllabus could decide to access the actual learning intake in a variety of methods. The game itself already helps identifying as it is played which are the concepts or terms unclear and recapitulated where needed. The trainer can also ask the class to pair up and explain to each other how the game works. The groups can also be asked to write in their own words what each part of the game stands for (using flipchart) or to actually draw the flow of communication when making a query between the parts as played in the game. Finally, it could be easy to implement a short quiz with multiple choices about the specific terms for example.

**ATTENDANCE REQUIREMENTS:**
Trainees have to actively interact in the game, therefore fully attend it from the start to end.

**Extension to the game to other topics can be found here:**

[**http://www.biotnet.org/training-materials/psicquic-game**](http://www.biotnet.org/training-materials/psicquic-game)

[**http://www.biotnet.org/training-materials/web-services-game**](http://www.biotnet.org/training-materials/web-services-game)
